# Vitamin D_3_ Loading Is Superior to Conventional Supplementation After Weight Loss Surgery in Vitamin D-Deficient Morbidly Obese Patients: a Double-Blind Randomized Placebo-Controlled Trial

**DOI:** 10.1007/s11695-016-2437-0

**Published:** 2016-11-12

**Authors:** Maria Luger, Renate Kruschitz, Christian Kienbacher, Stefan Traussnigg, Felix B. Langer, Gerhard Prager, Karin Schindler, Enikö Kallay, Friedrich Hoppichler, Michael Trauner, Michael Krebs, Rodrig Marculescu, Bernhard Ludvik

**Affiliations:** 1 Division of Endocrinology and Metabolism, Department of Internal Medicine III, Medical University of Vienna, Vienna, Austria; 2 Special Institute for Preventive Cardiology and Nutrition-SIPCAN Save Your Life, Salzburg, Austria; 3 Institute of Social Medicine, Centre for Public Health, Medical University of Vienna, Vienna, Austria; 4 Department of Medicine 1 and Karl Landsteiner Institute for Obesity and Metabolic Diseases, Rudolfstiftung Hospital, Juchgasse 25, 1030 Vienna, Austria; 5 Division of Gastroenterology and Hepatology, Department of Internal Medicine III, Medical University of Vienna, Vienna, Austria; 6 Division of General Surgery, Department of Surgery, Medical University of Vienna, Vienna, Austria; 7 Department of Pathophysiology and Allergy Research, Medical University of Vienna, Vienna, Austria; 8 Division of Internal Medicine, Krankenhaus der Barmherzigen Brüder Salzburg, Salzburg, Austria; 9 Clinical Institute for Medical and Chemical Laboratory Diagnostics, Department of Laboratory Medicine, Medical University of Vienna, Vienna, Austria

**Keywords:** Vitamin D, Vitamin D supplementation, Obesity, Gastric bypass, Omega-loop gastric bypass, Weight loss, Secondary hyperparathyroidism, Liver fibrosis

## Abstract

**Background:**

Bariatric patients often suffer from vitamin D deficiency (VDD), and both, morbid obesity and VDD, are related to non-alcoholic fatty liver disease. However, limited data are available regarding best strategies for treating VDD, particularly, in bariatric patients undergoing omega-loop gastric bypass (OLGB). Therefore, we examined the efficacy and safety of a forced vitamin D dosing regimen and intervention effects in liver fibrotic patients.

**Methods:**

In this double-blind, randomized, placebo-controlled trial, 50 vitamin D-deficient patients undergoing OLGB were randomly assigned to receive, in the first month postoperatively, oral vitamin D_3_ (≤3 doses of 100,000 IU; intervention group) or placebo as loading dose (control group) with subsequent maintenance dose (3420 IU/day) in both groups until 6-month visit.

**Results:**

Compared with control group, higher increase of 25(OH)D (67.9 (21.1) vs. 55.7 nmol/L (21.1); *p* = 0.049) with lower prevalence of secondary hyperparathyroidism (10 vs. 24 %; *p* = 0.045) was observed in intervention group. No (serious) adverse events related to study medication were found. The loading dose regimen was more effective in increasing 25(OH)D in patients with significant liver fibrosis while this was not the case for conventional supplementation (placebo with maintenance dose) (71.5 (20.5) vs. 22.5 nmol/L (13.8); *p* = 0.022; *n* = 14).

**Conclusions:**

Our findings indicate that a high vitamin D_3_ loading dose, in the first month postoperatively, with subsequent maintenance dose is effective and safe in achieving higher vitamin D concentrations in OLGB patients. Unexpectedly, it is more effective in patients with significant liver fibrosis which is of potentially high clinical relevance and requires further investigation.

**Electronic supplementary material:**

The online version of this article (doi:10.1007/s11695-016-2437-0) contains supplementary material, which is available to authorized users.

## Introduction

Vitamin D_3_ (cholecalciferol), derived both from dermal synthesis and dietary sources (e.g., oily fish and supplements), is first modified mainly in the liver by 25-hydroxylation to generate the main circulating form, 25-hydroxy vitamin D (25(OH)D). This is followed by 1α-hydroxylation to produce the active hormone, 1,25-dihydroxy vitamin D (1,25(OH)_2_D) mainly in the kidney and also at numerous extra-renal sites [1]. The vitamin D receptor (VDR) is expressed almost ubiquitously and regulates, upon ligand binding, the expression of over 200 genes [2]. In addition, non-VDR-mediated vitamin D actions have been identified in various tissues and this field of research is rapidly expanding [3]. It is therefore not surprising that the vitamin D endocrine system impacts the function of virtually every organ system in the human body. Vitamin D deficiency has been connected by mechanistic, epidemiologic, and clinical intervention studies with the pathogenesis of numerous diseases [3]. Obese patients are at higher risk of vitamin D deficiency compared with non-obese individuals [4], which seems to be best explained by the dilution of vitamin D and 25(OH)D in the expanded adipose compartment [5, 6]. More sophisticated pharmacokinetic studies are needed to clarify details such as the contribution of the adipose tissue itself to the metabolism of vitamin D. Obesity is also associated with non-alcoholic fatty liver disease (NAFLD), which comprises a disease spectrum ranging from simple steatosis to steatohepatitis, fibrosis, cirrhosis, and finally hepatocellular carcinoma and is the most prevalent chronic liver disease in industrialized countries [7]. Vitamin D insufficiency (25(OH)D <75 nmol/L) is associated with NAFLD independently from diabetes, insulin resistance, and metabolic syndrome [8]. Increased stages of liver fibrosis are associated with lower vitamin D concentrations in morbidly obese patients [9], suggesting a causal role of vitamin D deficiency in the pathogenesis of liver fibrosis. Vitamin D has even been proposed as a therapeutic agent for this condition [10].

Bariatric surgery is an effective method to treat obesity, (pre)diabetes [11, 12], and NAFLD [13] in morbidly obese patients and is associated with long-term weight loss and decreased overall mortality [14]. Due to gastric restriction and malabsorption following the gastric bypass procedure, 50–96 % of bariatric patients suffer from vitamin D deficiency, in addition to the obesity-related nutritional deficiencies [15–18].

Only limited data are available regarding the best strategies for treating vitamin D deficiency in bariatric patients. The clinical practice guidelines differ among scientific societies and often lack scientifically founded criteria. Therefore, there is need for high quality randomized controlled trials to provide reliable evidence for the recommendations on vitamin D supplementation in bariatric patients [19].

The primary aim of this study was to examine the efficacy and safety of a forced vitamin D dosing regimen (up to three oral loading doses in the first month postoperatively, intervention group) vs. conventional supplementation (placebo with following maintenance doses, control group) on parameters of vitamin D metabolism in morbidly obese, vitamin D-deficient patients undergoing omega-loop gastric bypass (OLGB) 6 months after surgery. In addition, given the high prevalence of liver fibrosis in these patients and the intricate role of vitamin D in this condition [9], we assessed markers of vitamin D metabolism in liver biopsies from the subgroup of patients affected by significant liver fibrosis.

## Subjects and Methods

The “Link Between Obesity and Vitamin D” (LOAD) study conducted from April 2014 to October 2015 in Vienna (Austria), was a 6-month double-blind, placebo-controlled, randomized clinical trial with a parallel-group design in bariatric patients. The effects of up to three oral vitamin D_3_ loading doses in the first month postoperatively followed by a maintenance dose (intervention group) was compared with placebo followed by a maintenance daily dose (control group) on 25(OH)D concentration and other vitamin D metabolism parameters. The details on design and the used materials and methods of the study have been previously published [20].

### Participant Recruitment and In- and Exclusion Criteria

Bariatric patients with following inclusion criteria were recruited: men and women aged 18–100 years with planned OLGB surgery, serum 25(OH)D concentrations of <75 nmol/L, and body weight <140 kg (due to body weight limitation of the dual energy X-ray absorptiometry (DEA)). Specific exclusion criteria included any other planned form of bariatric surgery than OLGB, hypo- and hypercalcemia, renal insufficiency (creatinine >133 μmol/L or glomerular filtration rate <50 mL min^−1^ 1.73 m^−2^), or primary hyperparathyroidism [20]. In- and out-patients of the Obesity Clinics at the Department of Internal Medicine III or the Department of Surgery in the General Hospital of Vienna were recruited between April 2014 and April 2015. All study procedures were reviewed and approved by the local Ethics Committee of the Medical University of Vienna (No. 1899/2013) and by the Austrian Competent Authority (No. LCM-718280-0001) and comply with the Declaration of Helsinki [21]. Moreover, the protocol was registered at clinicaltrials.gov (identifier: NCT02092376) and EudraCT (identifier: 2013-003546-16). All study participants provided signed informed consent. The study methods are in accordance with the Consolidated Standards of Reporting Trials (CONSORT) guidelines for reporting randomized trials [22].

Sample size calculation was based on previously published data from 50 bariatric patients who underwent OLGB [18]. On basis of an assumed 20 % dropout rate (including loss to follow-up), we estimated a total sample size of 50 patients (25 in each group) by using 80 % statistical power and a two-sided significance level of 0.05 to detect a 25(OH)D difference of 30 nmol/L (standard deviation, 35) between intervention and control groups after 6 months.

### Study Design and Randomization

After collecting baseline data, eligible patients were allocated to receive either vitamin D3 (intervention group) or placebo (control group) with a computer-generated randomization scheme in a 1:1 ratio, stratified by 25(OH)D, age, and gender level by a blinded study coordinator using the “Randomizer for Clinical Trials 1.8.1” [23]. Randomization was carried out during the baseline assessment, after the patient had signed the informed consent form. Everybody involved in the study was blinded to the randomization status. Allocation was performed by consecutively numbered dark bottles with either vitamin D_3_ (cholecalciferol diluted in medium-chain triglycerides: Oleovit D_3_ drops, Fresenius) or placebo (carrier oil of medium-chain triglycerides) loading dose, labeled with the randomization number, which were created and bottled by the in-house hospital pharmacy, so as to blind both study subjects and investigators.

### Vitamin D Dosing Regimen

In the field of pharmacology, a loading dose is an amount of drug designed to fill the central volume of distribution for a drug to a concentration that matches the final plateau concentration achieved with the maintenance dose. The purpose is to achieve this final plateau sooner than the four half-lives required, if the drug is simply administered at the maintenance dose rate [24]. For vitamin D_3_, the functional half-life within the body is in the range of 2 to 3 months [25]. Accordingly, the loading dose for the intervention group was calculated as the cumulative maintenance dose that was planned to be given through one functional half-life of vitamin D in the body (loading dose = (daily maintenance dose) × (60–90 days)). Moreover, it is safer not to take vitamin D at a dose beyond 100,000 IU at one time to allow vitamin D to clear from the circulation between each increment of the loading dose [1]. In that regard, the chosen loading dose of 300,000 IU vitamin D_3_ (cholecalciferol) was divided into three doses (each 100,000 IU) and given on day 1 or 2, 2 weeks, and 4 weeks postoperatively with subsequent administration of the maintenance dose until the 6-month visit in the intervention group [20]. The first loading dose was given on day 1 or 2 after surgery, followed by the second (2 weeks) and third administrations (4 weeks postoperatively) if 25(OH)D serum concentration remained below 75 nmol/L. The maximum loading dose for the intervention group was 300,000 IU. After the last loading dose, a maintenance dose of 3420 IU/day (approximately translating to 24,000 IU/week) was given. The control group received placebo as loading dose and subsequently the maintenance dose, the same way as the intervention group. A detailed illustration of the dosing regimen is depicted in the study protocol [20]. At each study visit after surgery, participants received a monthly supply of study medication (Oleovit D_3_ drops, Fresenius, containing 12.5 mL of cholecalciferol in medium-chain triglycerides carrier oil; 1 drop = 400 IU). Every week, the patients received instructions and further reminders via text messages to take the supplement (maintenance dose). The patients were instructed to return empty bottles at the monthly study visit.

### Assessment of Variables

At baseline (before randomization), age, sex, medical history (e.g., comorbidities, prescribed medication) were collected [20]. The following set of evaluations was obtained for each participant before surgery and 0.5, 1, 2, 3, 4, 5, and 6 month(s) postoperatively: height and body weight (measured with the calibrated scale seca mBCA 515), waist circumference measured with an inelastic tape, supplement use, and parameters of vitamin D metabolism (25(OH)D (nmol/L), 1,25(OH)_2_D (pg/mL), intact parathyroid hormone PTHi (pg/mL), and albumin-corrected calcium Ca (mmol/L)) [26]. Secondary hyperparathyroidism (SHPT) was defined as PTHi >65 pg/mL with simultaneous normal values for creatinine, calcium, and inorganic phosphate. Daily total energy (kcal), relative energy from protein (%), carbohydrate (%), fat (%), vitamin D (μg), and calcium (mg) intakes were calculated from 5-day food records 1 week before surgery (baseline) and 1, 3, and 6 month(s) after surgery. The computation of nutrient intake was carried out with the nutritional software nut.s science (dato Denkwerkzeuge, v1.29.34, Austria). Additionally, habitual sun exposure was assessed by a series of questions for which categorical response options were provided [27] such as “hours spent outside between 9 a.m. and 3 p.m.” during the week (h/week), during the weekend (h/weekend), “regular going to the tanning salon” (yes, no), and “using sunscreen” (yes, no). Furthermore, an average time spent outside was calculated (h/week). Season was defined as spring (March–May), summer (June–August), autumn (September–November), and winter (December–February). Supplementation adherence was reviewed by medication counts.

### Surgical Technique

All procedures were performed by the same surgical team using a laparoscopic approach. The OLGB is a simplified procedure that consists of a unique gastrojejunal anastomosis between a 30- and 40-mL-sleeve gastric pouch and a jejunal omega-loop of approximately 200 cm [28].

### Liver Biopsy and Histopathological Evaluation

Intraoperatively, fine-needle trucut biopsies were performed during the laparoscopic OLGB. All tissues were fixed in 10 % buffered formalin and embedded in paraffin. The three histochemically stained biopsies (hematoxylin and eosin, Chromotrop Anilinblue and Prussian blue iron stains) were analyzed and interpreted by two independent, experienced board-certified pathologists of the General Hospital Vienna, Austria. The histological scoring system NAFLD activity score (NAS; from 0 to 8) by Kleiner et al. [29] was used to evaluate the grade of steatosis (0–3), hepatocyte ballooning (0–2), lobular inflammation (0–3), and stage of fibrosis with a 4-point scale.

### Safety and Adverse Effects

The participants were interviewed after 2 weeks, 1, 2, 3, 4, 5, and 6 month(s) postoperatively for any signs or symptoms of vitamin D toxicity or other adverse events, including serious illness or hospitalizations.

### Statistical Analysis

The results are expressed as mean (standard deviation) for continuous and as percentages for categorical variables. In order to test for normal distribution, a visual test (histograms and box plots) was used and the Kolmogorov-Smirnov test was applied in addition. Statistical significance tests such as *t* test or Mann-Whitney *U* test, and Chi^2^ test were applied to assess differences between the intervention and control groups at baseline. The primary outcome variable 25(OH)D was analyzed according to the intention-to-treat (ITT) principle (including all randomized participants). To handle missing data, the multiple imputation (MI) method was used for the main analysis [30]. All secondary outcome variables were assessed by per-protocol analysis (PP; all persons included in the study without major protocol deviation). The associations of 25(OH)D and PTHi were assessed by multiple and single linear regression models with backward selection of variables at a *p* value threshold of 0.20 or variables entered in the model. We used repeated measure analysis of covariance (ANCOVA) using random error (linear mixed model) to assess the effect of time and the interaction for changes in parameters between the groups, by using different covariance structure models as appropriate and were adjusted for age, sex, and baseline values to supply an unbiased estimate of the mean group difference [31]. Moreover, a post hoc analysis with Bonferroni correction was used. Cohen’s *d* was used to quantify the effect of the treatment (effect size) based on the mean pre-postchange in the intervention group minus the mean pre-postchange in the control group, divided by the pooled pretest standard deviation [32, 33]. Estimates of the prevalence of SHPT and vitamin D sufficiency between the intervention and control groups over time were calculated using generalized estimating equation (GEE) with a logit link function for binary outcomes and unstructured covariance matrices. With this approach, we examined effects with time as repeated factor and group as between subject factor with prevalence of SHPT and vitamin D sufficiency (yes, no) as dependent variable, adjusted for age, sex, and baseline value. All statistical analyses were performed with IBM® SPSS® Statistics for Windows, v23 software (IBM Corporation, Armonk, New York, USA). *p* values <0.05 were considered statistically significant, and all tests were two sided.

## Results

### Study Recruitment and Follow-up

**Fig. 1:**
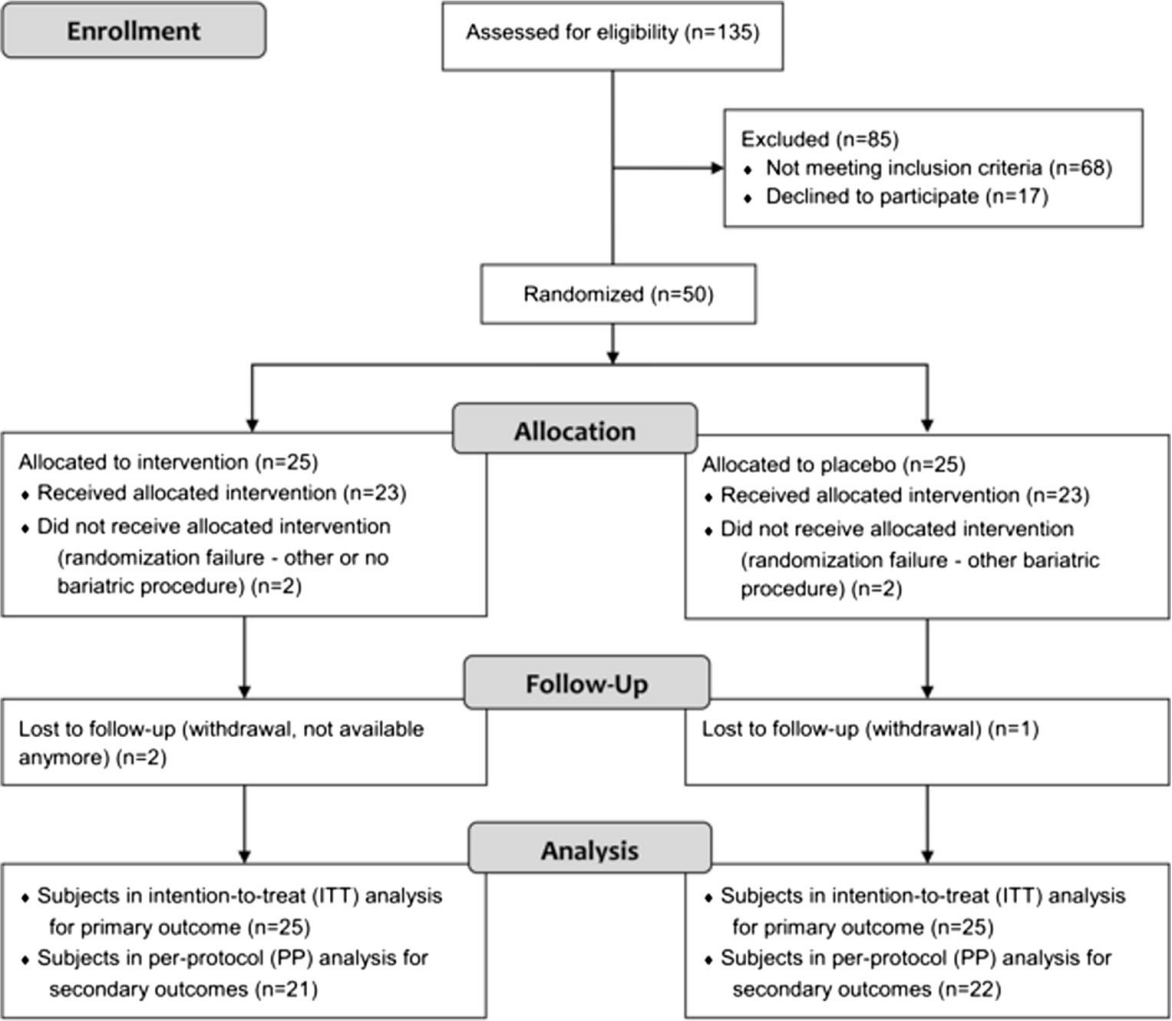
CONSORT flow chart of participant recruitment in the randomized controlled trial

Out of 67 eligible patients, 17 declined to participate (25 %) (Fig. 1). The remaining 50 patients were randomized and 25 each were allocated either to intervention or control group. In total, the dropout rate was 6 % (*n* = 3), 8 % (*n* = 2) in the intervention and 4 % (*n* = 1) in the control group. The final number of patients in the per-protocol analysis for secondary outcome measures was 21 in the intervention and 22 in the control group (Fig. 1). Enrollment took place continuously from April 2014 to April 2015, however, the number of patients randomly assigned to each group did not differ by season (*p* = 0.972).

Regarding the first, second, and third loading doses in the first month postoperatively, 100, 100, and 96 % of the participants took their assigned study drug (cholecalciferol) or placebo. Adherence to the subsequent maintenance dose was at 2, 3, 4, 5, and 6 months after surgery at 67, 70, 71, 63, and 61 % without statistically significant differences between the study groups.

### Baseline Characteristics

The baseline characteristics of randomized patients separately for the respective study groups are shown in Table 1 and in the supplementary material (Table S1; dietary intake). All patients had 25(OH)D concentrations below 75 nmol/L. At baseline, no statistically significant differences between intervention and control groups were observed, except for serum concentration of PTHi (*p* = 0.039). Serum 25(OH)D concentration correlated significantly with season (summer; *β* = 0.503, *p* < 0.001) and age (*β* = 0.386, *p* = 0.005).

**Table 1:** Patients’ characteristics at baseline between intervention and control groups

	Total (*n* = 50)	Intervention (*n* = 25)	Control (*n* = 25)
Age (years)	42.4 (12.7)	43.0 (12.6)	41.8 (13.0)
BMI (kg/m^2^)	43.8 (4.3)	44.6 (4.2)	42.9 (4.3)
Waist circumference (cm)	127.4 (10.6)	128.3 (9.7)	126.5 (11.5)
Number of drugs	5.8 (8.1)	5.8 (8.3)	5.8 (8.0)
Female (*n* (%))	40 (80)	20 (80)	20 (80)
Comorbidities			
Cardiovascular (*n* (%))	28 (56)	13 (52)	15 (60)
Diabetes mellitus (*n* (%)	13 (26)	9 (36)	4 (18)
Depression (*n* (%))	8 (16)	2 (8)	6 (24)
Significant liver fibrosis (*n* (%))^a^	14 (30)	9 (43)	5 (20)
Supplements intake			
Vitamin D (IU/day)^b^	252.3 (675.2)	130.3 (407.3)	374.4 (856.7)
Calcium (mg/day)^c^	360.0 (339.4)	600.0 (0.0)	120.0 (0.0)
Time outside (h/week)	13.7 (11.1)	13.7 (10.0)	13.8 (12.3)
Serum parameters			
25(OH)D (nmol/L)	39.0 (14.4)	38.8 (14.2)	39.3 (14.8)
1,25(OH)_2_D (pg/mL)	46.9 (16.2)	44.9 (14.2)	48.8 (17.9)
PTHi (pg/mL)	48.7 (14.3)	45.1 (14.7)	52.3 (13.3)*
Calcium (mmol/L)	2.3 (0.1)	2.3 (0.2)	2.3 (0.1)
Corr. Ca (mmol/L)	2.2 (0.1)	2.2 (0.1)	2.2 (0.1)

Note: Data are presented as mean (standard deviation) or percentages. *T* test or Mann-Whitney *U* test depending on distribution

*25(OH)D* 25-hydroxy vitamin D, *1,25-(OH)*_*2*_ *D* 1,25-dihydroxy vitamin D, *PTHi* parathyroid hormone intact, *Ca* calcium, *corr. Ca* corrected total calcium, *BMI* body mass index

*p < 0.05 (intervention vs. control)

^a^Significant liver fibrosis *F* ≥ 2

^b^Vitamin D via supplements before receiving the study drug of cholecalciferol

^c^ *n* = 2 received calcium supplementation (*n* = 1 intervention, *n* = 1 control)

### Change in Primary Outcome Variable

**Fig. 2:**
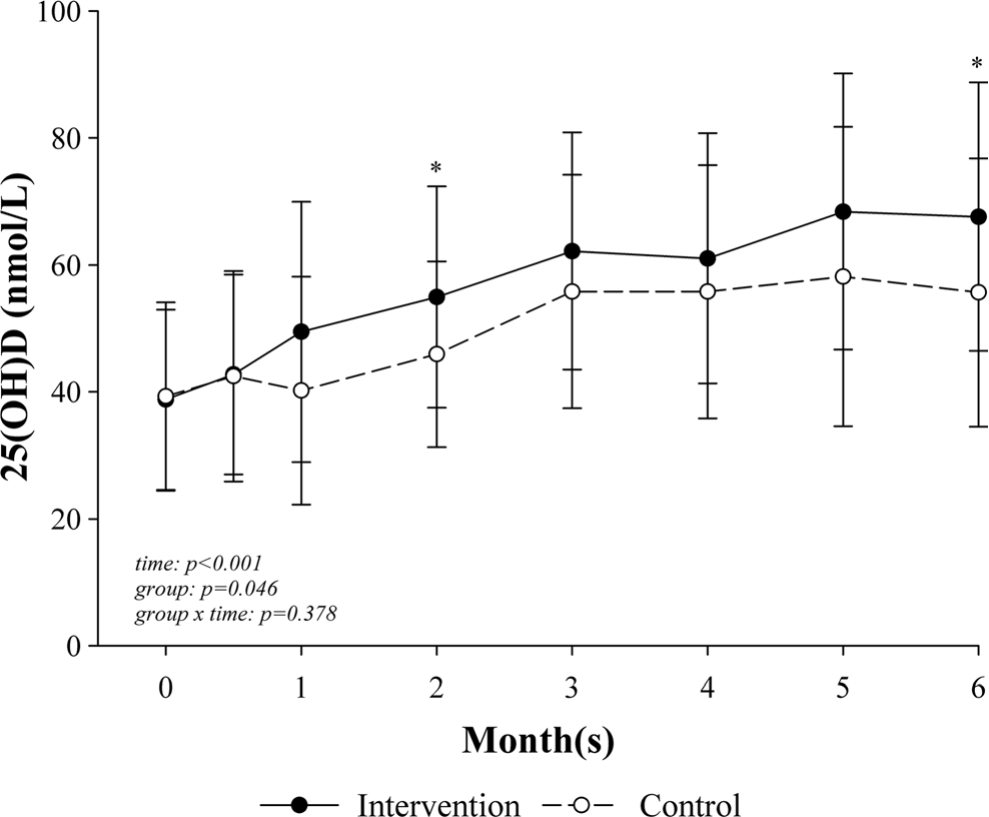
Change in serum 25-hydroxy vitamin D concentration (nmol/L) over the time between intervention and control groups. Note: *25(OH)D* 25-hydroxy vitamin D. Repeated measure analysis of variance and post hoc analysis with Bonferroni correction, adjusted for baseline value, season, age, and sex with intention-to-treat analysis: intervention (*n* = 25) and control (*n* = 25). *Error bars*: standard deviation; *p < 0.05 (intervention vs. control)

The significant change in serum 25(OH)D during the study is shown in Fig. 2. Vitamin D supplementation showed a significant increase in 25(OH)D over time (*p* < 0.001) and with a significantly higher 25(OH)D concentration in the intervention compared with the control group (*p* = 0.046). The difference between the groups was significantly different at 2 months (*p* = 0.031) and 6 months (*p* = 0.049) postoperatively. Moreover, the intervention group achieved a maximum 25(OH)D concentration of 75.7 nmol/L (standard deviation, 20.5; *C*_max_) at 4.7 months (1.6; *T*_max_) with an area under the curve (AUC) of 339.6 (standard deviation, 103.2) and 52 % showed a *C*_max_ within the normal range of >75 nmol/L over the time period. In comparison, the control group demonstrated a *C*_max_ of 67.5 nmol/L (20.6) at 4.2 months (1.3) with an AUC of 261.6 (81.7) and 41 % showed a normal *C*_max_ over the study duration. The AUC differed significantly between intervention and control groups (*p* = 0.009). In addition, the effect size Cohen’s *d* was 0.87 and can be considered a large effect size, according to Cohen [32].

By using generalized estimating equation, adjusted for age, sex, season, and baseline value, we found no significant difference in the estimates of the prevalence of vitamin D sufficiency (25(OH)D ≥75 nmol/L) between the intervention and control groups (*p* = 0.274), but an increase in the prevalence over time (*p* = 0.008): intervention vs. control: no difference at baseline; after 2 weeks, 2 vs. 1 %; 1 month, 5 vs. 3 %; 2 months, 6 vs. 3 %; 3 months, 24 vs. 15 %; 4 months, 23 vs. 13 %; 5 months, 40 vs. 34 %; and after 6 months, 34 vs. 22 %. Moreover, patients in the intervention group showed an adjusted odds ratio of 1.9 (95 % CI = 0.6, 5.9; *p* = 0.274) for vitamin D repletion compared with the control group.

### Change in Secondary Variables

Changes in BMI, waist circumference, intake of supplements (vitamin D and calcium), and serum parameters of vitamin D metabolism are shown in Table 2. Vitamin D intake via supplementation significantly changed over time from baseline (*p* < 0.001) between the groups (*p* < 0.001) with differences at 0.5, 1, and 2 months and showed a group and time interaction (*p* < 0.001), as the change over time differed between the groups. The activated serum vitamin D concentration, 1,25(OH)_2_D_3_, changed significantly over time (*p* = 0.012) and after 3 months differed between the groups (*p* = 0.050). Ca supplementation, serum PTHi, Ca, and corrected calcium concentrations showed no significant changes over time, between the study groups, and no group and time interactions. Dietary intake of energy, fat, carbohydrate, protein, calcium, and vitamin D is shown in Supplementary Material Table S2. The time spent outdoors (h/week; between 9 a.m. and 3 p.m. a day) was similar between the groups (intervention vs. control, 0.7 h/week (95 % CI = −3.3, 4.7), *p* = 0.717), over the time (−1.5 h/week (95 % CI = −6.6, 3.6), *p* = 0.345) and showed no group and time interactions (*p* = 0.877).

**Table 2:** Mean BMI, waist circumference, intake of supplements, and serum parameters after vitamin D supplementation and surgery in intervention and control groups

	2 weeks	1 month	2 months	3 months	4 months	5 months	6 months	*p*values^a^		
								Group	Time	Group × time
BMI (kg/m^2^)										
I	41.9 (4.1)	40.6 (4.3)	38.1 (4.2)	36.9 (3.9)	35.5 (3.7)	34.5 (3.6)	33.1 (3.9)	0.392	<0.001	0.217
C	40.8 (3.7)	39.4 (4.0)	37.4 (3.8)	35.4 (3.7)	34.2 (3.3)	32.3 (3.5)	31.1 (3.5)			
Waist circumference (cm)										
I	–	120.8 (6.9)	115.9 (6.3)	111.1 (8.6)	109.3 (8.8)	106.1 (8.3)	102.9 (8.8)	0.564	0.086	0.288
C	–	122.4 (10.1)	117.0 (10.4)	113.9 (10.8)	109.2 (10.1)	105.2 (8.9)	101.0 (9.2)			
Supplements intake										
Vitamin D (IU/day)										
I	7480.9 (1039.1)**	6905.4 (1544.9)**	5192.0 (2122.5)**	4136.3 (1997.3)	3240.3 (788.4)	3379.2 (615.6)	3299.5 (1012.5)	<0.001	<0.001	<0.001
C	489.1 (913.0)**	580.0 (918.5)**	3249.0 (1159.2)**	3790.4 (1291.1)	3381.2 (1448.8)	3472.3 (1019.1)	3445.1 (1316.4)			
Ca (mg/day)										
I	220.0 (316.2)	225.3 (315.3)	251.0 (337.3)	341.0 (500.9)	250.5 (328.7)	207.0 (268.3)	238.6 (319.1)	0.146	0.281	0.290
C	120.0 (0.0)	175.6 (235.7)	200.0 (254.6)	196.2 (248.8)	200.0 (254.6)	206.0 (254.1)	203.6 (242.5)			
Serum parameters										
1,25(OH)_2_D (pg/mL)										
I	55.0 (20.7)	61.3 (15.3)	59.1 (11.5)	69.6 (29.3)*	60.7 (11.0)	62.3 (12.0)	66.2 (16.8)	0.105	0.012	0.447
C	50.6 (11.9)	55.9 (14.7)	57.1 (16.5)	59.2 (12.6)*	60.2 (17.7)	60.0 (17.7)	60.5 (17.1)			
PTHi (pg/mL)										
I	51.0 (24.7)	48.2 (14.7)	49.8 (15.1)	50.9 (19.0)	50.4 (15.3)	48.3 (16.3)	47.6 (15.6)	0.364	0.367	0.952
C	58.7 (23.0)	58.9 (19.3)	63.2 (23.9)	61.0 (24.3)	60.5 (22.6)	58.3 (24.7)	54.4 (23.2)			
Ca (mmol/L)										
I	2.3 (0.1)	2.3 (0.1)	2.4 (0.1)	2.3 (0.1)	2.3 (0.1)	2.3 (0.1)	2.3 (0.1)	0.848	0.461	0.223
C	2.3 (0.1)	2.3 (0.1)	2.3 (0.1)	2.3 (0.1)	2.3 (0.1)	2.3 (0.1)	2.3 (0.1)			
Corr. Ca (mmol/L)										
I	2.2 (0.1)	2.2 (0.1)	2.2 (0.1)	2.2 (0.1)	2.2 (0.1)	2.2 (0.1)	2.2 (0.1)	0.600	0.313	0.365
C	2.2 (0.1)	2.2 (0.1)	2.2 (0.1)	2.2 (0.1)	2.2 (0.1)	2.2 (0.1)	2.2 (0.1)			

Note: Data are presented as mean (standard deviation); at 6 months, *n* = 21 in intervention and *n* = 22 in control group except in 25(OH)D which is the primary outcome variable

*I* intervention, *C* control, *BMI* body mass index, *vit. D* vitamin D, *Ca* calcium, *25(OH)D* 25-hydroxy vitamin D, *1,25-(OH)*_*2*_ *D* 1,25-dihydroxy vitamin D, *PTHi* parathyroid hormone intact, *corr. Ca* corrected total calcium

*p < 0.005; **p < 0.001 between groups

^a^Repeated measure analysis of variance and post hoc analysis with Bonferroni correction, adjusted for baseline values, age, and sex

### Impact of Vitamin D Supplementation in Patients with Liver Fibrosis

**Fig. 3:**
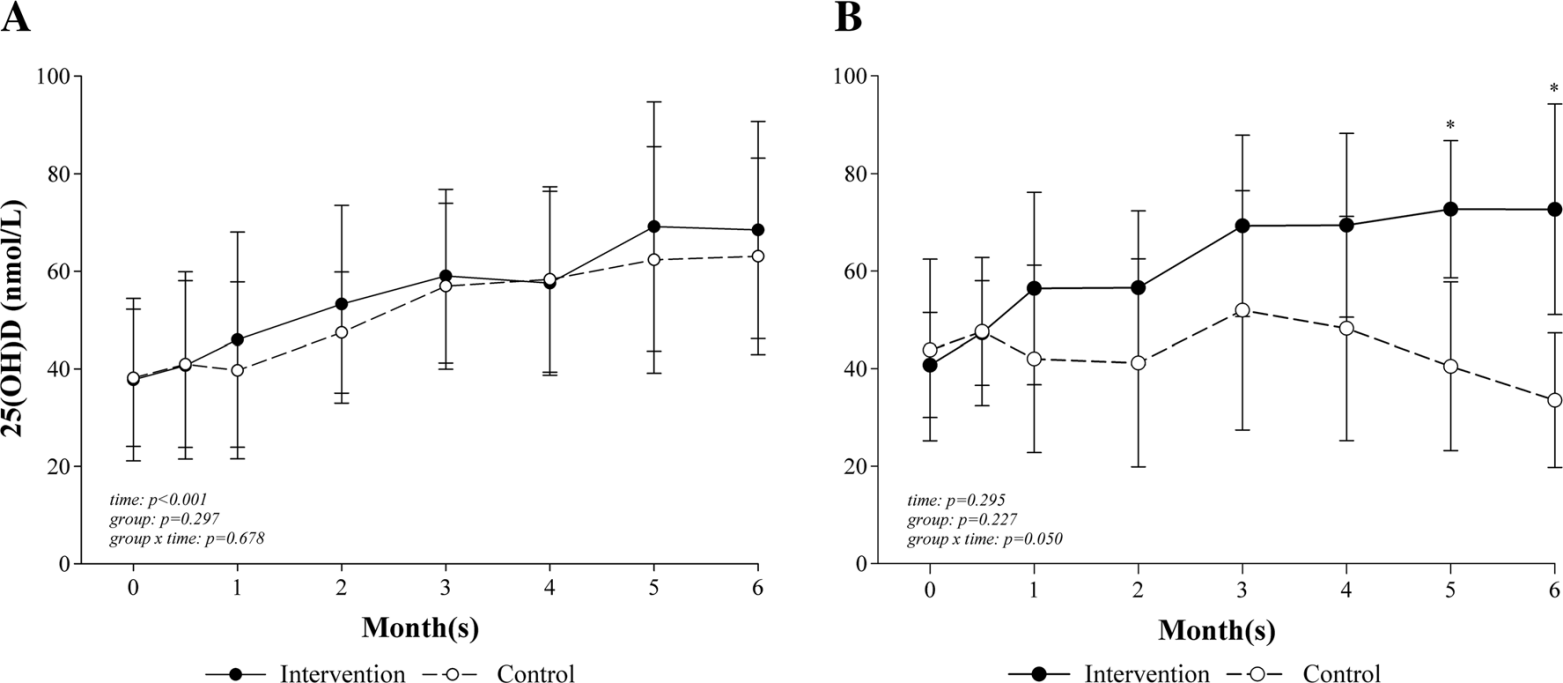
Change in serum 25-hydroxy vitamin D concentration (nmol/L) over the time between intervention and control groups in patients without (**a**) and with significant fibrosis (**b**). Note: *25(OH)D* 25-hydroxy vitamin D. Repeated measure analysis of variance and post hoc analysis with Bonferroni correction, adjusted for baseline value, vitamin D dose, season, age, and sex and intention-to-treat analysis: intervention (*n* = 25) and control (*n* = 25). Significant fibrosis = *F* ≥ 2, *n* = 14. No significant fibrosis = *F* ≤ 1, *n* = 29. *Error bars*: standard deviation; *p < 0.05 (intervention vs. control)

Figure 3 shows 25(OH)D concentrations in patients without (*F* ≤ 1; *n* = 29; Fig. 3a) and with (*F* ≥ 2; *n* = 14; Fig. 3B) significant liver fibrosis. Patients with fibrosis showed a significant group and time interactions (*p* = 0.050) with significant differences at 5 months (*p* = 0.050) and 6 months (*p* = 0.022) after surgery, as the concentration in the intervention group (*n* = 9) increased, while it decreased in the control group (*n* = 5). In comparison, 25(OH)D concentrations in patients without fibrosis significantly increased over the time (*p* < 0.001), but the increase was similar between the groups (intervention: *n* = 12, control: *n* = 20; *p* = 0.297).

### Parathyroid Hormone Suppression

**Fig. 4:**
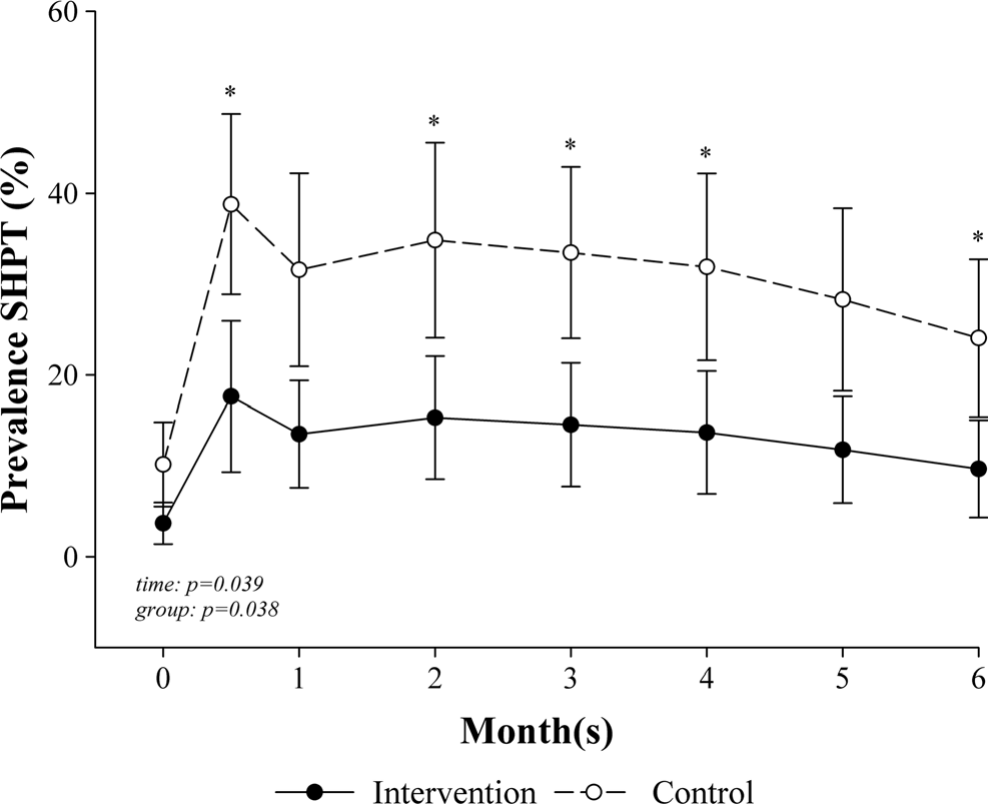
Estimates of the prevalence of secondary hyperparathyroidism over the time between intervention and control groups. Note: *SHPT* secondary hyperparathyroidism. Generalized estimating equations (GEE) with a logit link function for binary outcomes, adjusted for baseline value and sex; *bars* represent standard error; at 6 months, *n* = 21 in intervention and *n* = 22 in control group; *p < 0.05 (intervention vs. control)

We observed a significant suppression of PTHi in the intervention but not in the control group. The prevalence of SHPT decreased over the time (*p* = 0.039) and differed significantly between the groups (*p* = 0.038) with differences at 2 weeks (*p* = 0.024), 2 months (*p* = 0.046), 3 months (*p* = 0.032), 4 months (*p* = 0.042), and at the end of the study (6 months; *p* = 0.045; Fig. 4). Moreover, patients in the intervention group had an odds ratio of 0.3 (95 % CI = 0.1, 0.9; *p* = 0.038) for SHPT compared with the control group. PTHi was associated with 25(OH)D concentrations (*β* = −0.393, *p* < 0.001) and BMI (*β* = 0.164, *p* = 0.035) over the time course, adjusted by age and sex.

### Safety

No serious adverse events related to vitamin D_3_ supplementation were observed between the groups. No individual in either group had evidence of hypercalcemia (>10.5 mg/dL) during the study period (before and 6 months after surgery).

There were two serious adverse events over the whole study period: serious illnesses (myocardial infarction) before the bariatric procedure with consequent cancelation of the scheduled operation and after surgery (liver hematoma) as a consequence of liver biopsy during surgery. Therefore, no serious adverse event could be related to the study medication of cholecalciferol as both occurred before administration.

## Discussion

The aim of this study was to examine the efficacy and safety of a forced vitamin D dosing regimen vs. conventional supplementation on parameters of vitamin D metabolism in bariatric patients undergoing OLGB during 6 months. We have shown recently that 80 % of patients remained vitamin D deficient following OLGB, despite taking individually adjusted vitamin D_3_ supplementation of 200–3000 IU/day [18]. Thus, the current study adds new evidence regarding postoperative prevention and treatment of vitamin D deficiency in bariatric patients. This is particularly important for patients undergoing OLGB, which is a rather new bariatric procedure with higher weight loss compared with the Roux-en-Y gastric bypass [34].

Our current trial led to four main outcomes: *First*, due to the loading doses in the first month postoperatively and subsequent supplementation with maintenance doses, we observed a significantly higher increase in 25(OH)D concentration and AUC during the study period in the intervention group compared with the control group. *Second*, the intervention group had a significantly lower prevalence and odds rate of SHPT compared with the control group. PTHi was inversely associated with 25(OH)D concentrations over the time course. This could imply that higher vitamin D concentrations are needed to prevent SHPT which is associated with preoperative obesity and postoperative malabsorption [35]. *Third*, this vitamin D supplementation regimen is safe as we observed no (serious) adverse events related to the study medication. Serum calcium concentration remained stable throughout the study in each group despite the increasing dietary calcium intake. *Fourth*, the loading dose regimen was effective in increasing 25(OH)D concentrations in patients with significant liver fibrosis while this was not the case for the conventional dosing regimen.

To our knowledge, only one randomized controlled trial with higher doses of vitamin D in bariatric patients was published [35]. The authors randomized patients after Roux-en-Y gastric bypass to three doses of cholecalciferol (800, 2000, and 5000 IU/day). After 12 months, 44, 78, and 70 % of the patients reached sufficient vitamin D concentrations [35]. All other studies have either a retrospective cross-sectional design, are pilot studies, or have prospective but non-randomized study design [36–38]. Thus, the results of our study represent the best available evidence for an effective vitamin D substitution following gastric bypass. However, even in the intervention group, there was a high prevalence of low 25(OH)D concentration (<75 nmol/L) 6 months after surgery (66 % in the intervention group vs. 78 % in the controls). As the majority of the patients (96 %) required all three loading doses, it can be suggested that patients with vitamin D deficiency and planned OLGB should receive the vitamin D_3_ loading dose already before surgery. Nevertheless, this regimen needs to be evaluated in future prospective studies. Moreover, considering the use of the supplement matrix, a systematic review showed a higher vitamin D bioavailability by using an oil-soluble vehicle which led to a larger increase in serum 25(OH)D levels compared with non-oily vehicles in healthy individuals [39]. A recent randomized controlled trials also confirmed this finding [40]. Furthermore, while the used type of vitamin D_3_ supplementation in our study has not been examined in bariatric patients until now, it was assessed in a similar manner in obese children, in nursing home patients, patients with hip fracture surgery, and with inflammatory/autoimmune rheumatic diseases [41–44]. These studies have also shown superior effects of vitamin D_3_ loading dose supplementation similar to our study.

In the current study, the loading dose regimen with vitamin D_3_ was the only one effective in increasing 25(OH)D concentrations in patients with significant liver fibrosis over the 6-month study period in contrast to the conventional regimen using the same maintenance dose. This is an unexpected and remarkable finding. It is well established that serum 25(OH)D concentrations are significantly reduced in various types of chronic liver disease [8, 9, 45]. Several responsible mechanisms have been identified, whose relative contributions seem to vary depending on the etiology and the stage of the liver disease. Reduced vitamin D absorption in the terminal ileum due to decreased availability of bile acids has been implicated [46]. Significantly reduced expression of the hepatic vitamin D 25-hydroxylases CYP2R1 and CYP27A1 was described in chronic hepatitis C. In the same immunohistochemic study, reduced expression of these enzymes was also found in NASH; however, it did not reach statistical significance in this condition [47]. Serum concentrations of vitamin D-binding protein (VDBP) were found to be significantly decreased in NAFLD and to display a significant inverse correlation with the stage of fibrosis [48]. As VDBP is the main vitamin D carrier in blood, a decrease in its concentration would appear a likely contributor to lower total 25(OH)D concentration. Finally, genetic studies revealed an association of genetic variants in the vitamin D system with liver stiffness, suggesting a causal role in the pathogenesis of liver fibrosis [49]. This is also supported at the mechanistic level by evidence that vitamin D has metabolic, anti-inflammatory and anti-fibrotic effects on hepatocytes and non-parenchymal hepatic cells in NAFLD [50]. In that regard, vitamin D has been proposed as a potential therapeutic option for liver fibrosis [10]. Nevertheless, very few studies have been performed to evaluate the effects of vitamin D supplementation on liver fibrosis [51]. The significantly divergent time courses of 25(OH)D concentrations between loading dose and control group among patients with liver fibrosis over a period of 6 months in our present study is hard to explain by pharmacokinetic considerations alone. Therefore, although *n* = 14 is, of course, not sufficient to draw any reliable conclusions, it is tempting to attribute them to some pharmacologic effects of the initial megadoses of up to 3 × 100,000 units vitamin D_3_ in the intervention group, likely involving the described mechanisms [10] and presumably able to overcome some of the disturbances of the hepatic vitamin D metabolism associated with liver fibrosis. If confirmed, this finding would impact the design of future studies addressing the therapeutic utility of vitamin D in liver fibrosis.

Our study has some limitations: First, the sample size is rather small, although, it was based on the sample size calculation taking into account differences of serum 25(OH)D concentrations at 6 months between intervention and control groups. As we were able to find significant differences between the study groups regarding the primary outcome variable, the sample size can be regarded as sufficient. Second, our study included a high percentage of women (80 %); however, this is very common in bariatric patients. Third, underreporting of food-intake has been frequently observed in bariatric patients [52]. To minimize this phenomenon, our patients received dietary counseling by a registered dietician before surgery to keep a low-energy and low-carbohydrate diet and the dietary records were documented 1 week before surgery. All these facts might have contributed to the reduced preoperative energy intake.

Besides the significant and relevant findings, the double-blind, randomized, placebo-controlled study design and the close monitoring during the first 6 months postoperatively are further strengths of our study. Moreover, liver fibrosis has been biopsy proven as the current gold standard for the assessment of liver disease. This study provides detailed pre- and postoperative data of patients undergoing OLGB as a rather new bariatric procedure.

In conclusion, our findings indicate that starting the vitamin D_3_ supplementation with a high loading dose in the first month postoperatively followed by maintenance dose is effective and safe in achieving higher vitamin D concentrations in vitamin D-deficient bariatric patients receiving OLGB. Additionally, this dosing regimen appears to be the only one effective in patients with significant liver fibrosis. This is a remarkable finding of potentially high clinical relevance and requires further investigation. Larger studies regarding vitamin D supplementation in patients with liver fibrosis and a potential beneficial effect on this condition are warranted as both, morbid obesity and vitamin D deficiency are related to NAFLD.

## Electronic Supplementary Material

ESM 1 (DOCX 17 kb)
