# Supplementary material for: Vitamin D3 Loading Is Superior to Conventional Supplementation After Weight Loss Surgery in Vitamin D-Deficient Morbidly Obese Patients: a Double-Blind Randomized Placebo-Controlled Trial
Source: Obes Surg. 2016 Nov 12;27(5):1196–207. doi: 10.1007/s11695-016-2437-0 (PMC5403855; doi:10.1007/s11695-016-2437-0)
Supplement: Supplementary file 1 — (DOCX 17 kb) [file 11695_2016_2437_MOESM1_ESM.docx]

Supplementary materials

Table S1 Patients’ characteristics at baseline between intervention and control group

| **Dietary intake** ^a^ | **Total** (n=50) | | **Intervention** (n=25) | | | **Control** (n=25) |
| --- | --- | --- | --- | --- | --- | --- |
| Energy intake (kcal/d) | 1582.3 (628.8) | | 1810.0 (713.1) | | | 1354.7 (446.5)* |
| Relative energy from fat (%) | | 39.6 (7.7) | | 38.3 (8.6) | 40.9 (6.6) | |
| Rel. energy from carbohydrate (%) | | 40.7 (7.1) | | 40.1 (8.2) | 41.4 (5.9) | |
| Relative energy from protein (%) | | 19.9 (4.6) | | 20.5 (5.0) | 19.2 (4.2) | |
| Dietary calcium intake (mg/d) | 695.8 (380.7) | | 742.3 (484.6) | | | 649.4 (246.5) |
| Dietary vitamin D intake (IU/d) | 92.0 (80.0) | | 76.0 (72.0) | | | 104.0 (88.0) |

*Note: Data are presented as mean (standard deviation); ^a^ n=30 provided dietary protocols with 15 in intervention and 15 in control group; *p<0.05 (intervention vs. control)*

Table S2 Mean dietary intake after surgery in intervention and control group

| **Dietary intake** | | **1 month** ^a^ | **3 months** ^b^ | **6 months** ^c^ | **p-values ^+^** | | |
| --- | --- | --- | --- | --- | --- | --- | --- |
|  |  |  |  |  | Group | Time | Group x Time |
| Energy intake (kcal/d) | I | 830.1 (384.6) | 1141.3 (499.0) | 1174.9 (571.7) | 0.024 | 0.000 | 0.631 |
|  | C | 688.4 (308.1) | 905.2 (295.2) | 909.5 (404.9) |  |  |  |
| Relative energy from fat (%) | I | 33.0 (7.2) | 37.0 (6.3) | 34.7 (5.4) | 0.467 | 0.002 | 0.201 |
|  | C | 33.4 (8.7) | 31.4 (10.0) | 32.6 (8.8) |  |  |  |
| Rel. energy from carbohydrate (%) | I | 46.1 (8.4) | 43.6 (5.3) | 44.3 (4.3) | 0.969 | 0.164 | 0.906 |
|  | C | 45.4 (10.7) | 45.1 (10.6) | 42.7 (12.4) |  |  |  |
| Relative energy from protein (%) | I | 21.7 (8.2) | 20.0 (4.2) | 22.4 (4.8) | 0.579 | 0.307 | 0.781 |
|  | C | 22.2 (6.1) | 22.2 (11.7) | 25.1 (14.7) |  |  |  |
| Dietary calcium intake (mg/d) | I | 510.2 (220.5) | 633.2 (290.4) | 696.4 (350.3) | 0.872 | 0.185 | 0.709 |
|  | C | 501.1 (474.4) | 527.1 (450.7) | 955.6 (1310.3) |  |  |  |
| Dietary vitamin D intake (IU/d) | I | 70.1 (65.4) | 46.9 (25.9) | 43.9 (29.5) | 0.871 | 0.009 | 0.544 |
|  | C | 63.6 (73.4) | 30.8 (16.6) | 34.1 (21.7) |  |  |  |

*Note: Data are presented as mean (standard deviation); ^a^ n=25 provided dietary protocols with 12 in intervention and 13 in control group; ^b^ n=24 provided dietary protocols with 14 in intervention and 10 in control group; ^c^ n=22 provided dietary protocols with 12 in intervention and 10 in control group; ^+^ Repeated measures analysis of variance and post-hoc analysis with Bonferroni correction, adjusted for age and sex; I: Intervention; C:* Control*; *p<0.05 (intervention vs. control)*
